# Qualitative study to explore which components of home-based primary care may reduce emergency department visits among older adults

**DOI:** 10.1136/bmjopen-2026-118687

**Published:** 2026-09-11

**Authors:** Hanna Bring, Monica Bergqvist, Lars L Gustafsson, Karin Modig, Pia Bastholm-Rahmner, Katharina Schmidt-Mende

**Affiliations:** 1 Department of Neurobiology, Care Sciences and Society, Division of Family Medicine and Primary Care, Karolinska Institutet, Huddinge, Sweden; 2 Academic Primary Care Centre, Stockholm, Sweden; 3 Department of Neurobiology, Care Sciences and Society, Division of Nursing, Karolinska Institutet, Huddinge, Sweden; 4 Division of Clinical Pharmacology, Department of Laboratory Medicine, Karolinska University Hospital, Karolinska Institutet, Stockholm, Sweden; 5 Institute of Environmental Medicine, Unit of Epidemiology, Karolinska Institutet, Stockholm, Sweden

**Keywords:** Emergency Service, Hospital, Frail Elderly, Primary Health Care, Physicians

## Abstract

**Abstract:**

**Objective:**

Home-based primary care has been suggested to reduce emergency department visits among vulnerable older adults but how it should be organised to achieve this effect is unclear. This study aimed to increase the understanding of how older adults receiving home-based primary care and their family caregivers’ experience care during the period prior to an emergency department visit and to identify components of home-based primary care delivery that could help reduce such visits.

**Design:**

Qualitative study using semi-structured interviews with older adults receiving home-based primary care who had recently visited the emergency department, along with their family caregivers. Interviews were analysed using inductive thematic analysis.

**Setting:**

Interviews were conducted in the homes of informants receiving home-based primary care in Region Stockholm, Sweden, 2022–2023.

**Participants:**

Fourteen adults aged ≥65 years receiving home-based primary care who had visited an emergency department within the past 3 months were interviewed; seven interviews also included a family member.

**Results:**

An overarching theme, *Trust as a prerequisite for timely emergency assessments in home-based primary care*, describes how patient trust–in home-based primary care in general and in specific individual healthcare professionals–seems important in reducing emergency department visits, especially when patients experience symptoms of uncertain urgency or gradual onset. Four subthemes describe how primary care may build trust: through (1) access to care, (2) medical competence, (3) respect and relationship and (4) coordination and teamwork.

**Conclusion:**

Trust in both home-based primary care in general and in individual healthcare professionals is crucial in reducing emergency department visits among older adults receiving home-based primary care. Trust may be strengthened by accessible and competent care, familiarity with both general practitioners and the responsible nurse and working as a collaborative team.

**STRENGTHS AND LIMITATIONS OF THIS STUDY**

- The qualitative design using in-depth, semi-structured interviews enables detailed exploration of informants’ experiences.
- Purposeful sampling across socioeconomically diverse primary care practices contributes to variation in perspectives.
- Interviews were conducted up to 3 months after the emergency department visits, which may have affected informants’ recall of events.
- Informants were identified by home-based primary care staff, which may have influenced participant selection and the perspectives represented.
- Informants unable to provide informed consent or participate in an interview due to cognitive impairment were excluded, which may limit the transferability.

## Background

 Vulnerable older adults are more likely to visit the emergency department (ED) than the general population, and such visits are associated with complications, stress and mortality.^1^ About one-fourth of ED visits by older adults may be avoidable, although estimates vary across studies and countries.^2^ It remains unclear which strategies are most effective in reducing ED visits in this patient group, but pro-active interventions in primary care (PC) appear to be more effective than hospital-based approaches.^3^

Home-based primary care (HBPC)–also referred to as home healthcare in Scandinavian countries (Box 1)–has in the US shown potential to reduce ED visits among vulnerable, older adults with difficulties leaving their home due to illness or functional limitations.^3 4^ Although there is no widely recognised definition of HBPC, it generally includes interdisciplinary teams delivering long-term PC in the patient’s home.^4^ However, most studies on HBPC are from the US, and the experiences from universally funded healthcare systems have been less frequently studied.^4 5^ In Sweden, Stockholm is the only region which practices HBPC, with PC practices fully responsible for organisation of home healthcare. In other regions responsibility for home healthcare is shared with municipalities and more closely linked to home care (Box 1). The population receiving HBPC in Stockholm consists of individuals with significant functional limitations and a need for support from healthcare services due to difficulties leaving their homes.^6^ More than half of adults receiving both home care and HBPC have cognitive impairment, and approximately 85% require assistance with at least one activity of daily living, indicating a high level of vulnerability in this population.^6 7^

Box 1 **Organisation of home healthcare and home care in Stockholm, Sweden**

Healthcare in Sweden is universally funded and organised in 21 independent regions funded by taxes.^33^ Patients can receive different types of support in their homes. Home care provides assistance with activities of daily living such as personal hygiene and housekeeping, and is organised and funded by 290 municipalities.^33^ Home healthcare provides regular medical care such as drug administration and wound care. In Stockholm, home healthcare is delivered by general practitioners, district nurses and nurse assistants in primary care (defined as home-based primary care in this study) and initiated if the patient needs treatment or drug administration in the home at least 2 times/month. Advanced home healthcare is offered if patients need more complex treatments at home, such as intravenous medications.^33^

To better understand how HBPC should be provided to reduce ED visits, patient perspectives are essential.^8^ However, most previous research on patient perspectives on causes of ED visits has focused on older adults from the general population.^9–11^ The experiences of older adults with a high degree of functional and cognitive impairment, such as those receiving HBPC,^6^ are less well understood, despite their greater care needs and frequent contact with healthcare providers. Based on existing literature on HBPC, interdisciplinary teamwork, care coordination, and rapid at-home assessment have been suggested as potentially important components in reducing ED visits.^3 5^ Drawing on this knowledge and clinical experience of HBPC in Stockholm, we expected that, in particular, limited access to timely in-home assessment might be an important factor influencing care-seeking prior to ED visits.

Given the limited conceptual clarity in this specific context, the study adopted an exploratory qualitative design, allowing informants’ experiences to guide the analysis. The aim of this study is to increase the understanding of how older adults receiving HBPC and their family caregivers experience care during the period prior to an ED visit and to identify components of HBPC delivery–organisational, relational and clinical features of care–that could help reduce such visits.

## Materials and methods

### Study design

This is a qualitative study with semi-structured interviews analysed using inductive thematic analysis.^12^ Thematic analysis was chosen, as the aim was to get a rich description of informants’ experiences. We applied a constructivist framework, recognising that knowledge is individually constructed and shaped by both the informants’ and the researchers’ previous experiences.^13^

### Sampling and recruitment

To ensure geographical and socioeconomic diversity, we recruited informants from different parts of the Swedish capital region, Stockholm. PC practices with at least 20 older adults receiving HBPC were approached (n=171 out of a total of 221) to recruit older adults receiving HBPC. Inclusion criteria were age 65 years and older, at least one ED visit during the preceding 3 months, sufficient communication skills in Swedish or English and adequate health and cognitive ability to participate in an interview of approximately 1 hour.

HBPC staff identified 20 patients enrolled in HBPC as potential informants. Fourteen patients registered with 11 PC practices were interviewed in their homes. Patients could invite a family member or other support person to participate in the interview, and seven chose to do so, resulting in a total of 21 informants. Although inclusion was not restricted to individuals with caregiving roles, all accompanying persons were spouses or adult children and were involved in the patient’s care to varying degrees. For clarity, these informants are referred to as family caregivers in the results and discussion. Using the Care Need Index^14^ as a measure of socioeconomic status, informants were recruited from practices representing low (n=4), medium (n=6) and high (n=4) socioeconomic strata (table 1).

**Table 1:** Characteristics of the 14 interviews and 21 informants

Characteristic		Values
Interview length, minutes (mean (min-max))		45 (18–79)
Informants receiving home-based primary care		14
Gender (%)	Male	10 (71)
	Female	4 (29)
Age, years (mean (min-max))		83 (74–89)
Born in Sweden (%)	Yes	11 (92)
	No	1 (8)
Participating family caregivers		7
Gender (%)	Male	1 (14)
	Female	6 (86)

### Ethical considerations

This study conforms to the World Medical Association Declaration of Helsinki^15^ and was approved by the Swedish Ethical Review Authority (Dnr 2022-02692-01). To minimise the risk of harm to informants, HBPC staff who had personal knowledge of the patients initially assessed their suitability for participation. Subsequently, adults receiving HBPC and family caregivers were contacted by the researchers and provided with written and oral information about the study. Written informed consent was obtained from all informants. At the time of the interview, informants were provided with additional information about the study and given the opportunity to ask questions. Older adults receiving HBPC often experience cognitive impairment.^6^ Individuals with advanced forms of cognitive impairment were not invited to participate if they were judged unable to give informed consent. Participation was voluntary, and informants could withdraw at any time. The researchers were prepared to interrupt or terminate interviews if informants experienced distress; however, this did not occur. Informants received a gift certificate valued at 200 SEK (approximately 15 British pounds) as compensation for their participation.

### Patient and public involvement statement

Patients or the public were not involved in the design, conduct, or reporting of this study. However, the findings will inform the development of a PC intervention, for which patients and the public are actively involved in the co-design process.

### Data collection

An interview guide with open-ended questions was used (online supplemental additional file 1). The interview guide was developed collaboratively by all co-authors, based on the study aims, previous research and clinical experiences. The main questions used in the interview guide were:

- *Describe your healthcare contacts and how you experience them?*
- *What happened the day you last visited the ED?*
- *Do you think your ED visit could have been avoided, and if so, how?*

Two pilot interviews were conducted by co-author PBR in early 2022 and were included in the analysis. Following the pilot interviews, the interview guide was revised to place greater emphasis on the informants’ experience during the period before the ED visit. Subsequent interviews were conducted by HB and IL (see Acknowledgements) between November 2022 and September 2023. PBR is an experienced qualitative researcher. HB and IL have experience working with vulnerable older adults and were coached in qualitative interview techniques by PBR and MB. During interviewer coaching and preparation, the interviewers reflected on their interviewing technique and how their previous clinical experience and professional background could influence interactions with informants. This reflexive process was used to increase awareness of potential preconceptions. Interviewers had no previously established relationship with informants. All interviews were audio recorded and transcribed verbatim. Field notes were taken during some, but not all interviews. The field notes were read through during analysis to further inform the interpretation of data and analysis.

The concept of information power was used to determine the sample size.^16^ Data collection was concluded after including 14 interviews, considering the study’s narrow aim and the richness and relevance of the information obtained.

### Data analysis

Inductive thematic analysis was conducted, following the six-step methodology outlined by Braun and Clarke.^12^ HB, PBR and MB read all transcripts to become familiar with the data, made notes on patterns and discussed their observations. HB conducted the coding in NVivo (NVivo qualitative data analysis software, QSR International Pty Ltd, V.14), identifying similarities in the data and patterns of meaning without applying pre-existing theories or coding frameworks. After initial coding, the codes were examined and grouped into provisional themes based on shared meaning. For an example of coding, see table 2. Finally, HB, PBR, MB and KSM discussed and refined the salient patterns to enhance the depth of the analysis. These discussions were intended to support reflexive engagement with different interpretations and strengthen the analytic richness.^17^ HB was the main responsible researcher for the analysis and held final responsibility for the interpretation of the data, including in cases of differing analytic perspectives. Informants were not invited to provide feedback on the findings.

**Table 2:** Example of the process of data analysis

Quote	Code	Subtheme	Theme
P: The only thing is that they (the PC practice) might call back 2 hours later, but that is a different matter. Now they have started a system where, if you are over 75, you can call between 11 and 12 and speak directly to someone, which is very good.	You can just call or go to the PC practice	Accessible HBPC offering alternative care pathways to ED visits	Trust as a prerequisite for timely emergency assessments in HBPC
P: But if this doctor doesn’t even understand that I have back pain and instead says that I have pain in my knees–how is it possible to make a diagnosis then? (…) IC: If I am dependent on him and he is inadequate, then how is that supposed to work?	Incompetence in HBPC	Medical competence as a foundation for trust in HBPC	
P: And when you do have that contact, I at least want that person - the nurse or the doctor - not to see me as just the next case. I would really prefer there to be continuity.(…)Because it is always very difficult when you need to seek care and have to repeat the same story maybe five times to different people.	Continuity and relationships are important	The importance of continuity and respect in patient-provider relationships	
IC: It is really just a dream, but one could imagine that the district nurses would receive the information and respond by saying that they will take it back to the PC practice.	Fragmented care	HBPC’s responsibility is to coordinate care through teamwork and follow-up	

ED, emergency department; HBPC, home-based primary care; IC, family caregiver; PC, primary care.

### Trustworthiness

Reporting of this study adheres to Consolidated criteria for Reporting Qualitative research (COREQ) guidelines.^18^ The research team included members with expertise in nursing, general practice and behavioural science, bringing multiple perspectives to increase credibility. To increase transferability, we provide descriptions of the context and informants. Confirmability was strengthened through ongoing discussions on reflexivity during analysis, considering how disciplinary backgrounds and assumptions shaped interpretations. For example, HB reflected on how her experience as a GP working with older adults in HBPC could influence data interpretation, while discussions within the research team helped challenge assumptions and broaden perspectives.

## Results

**Figure 1:**
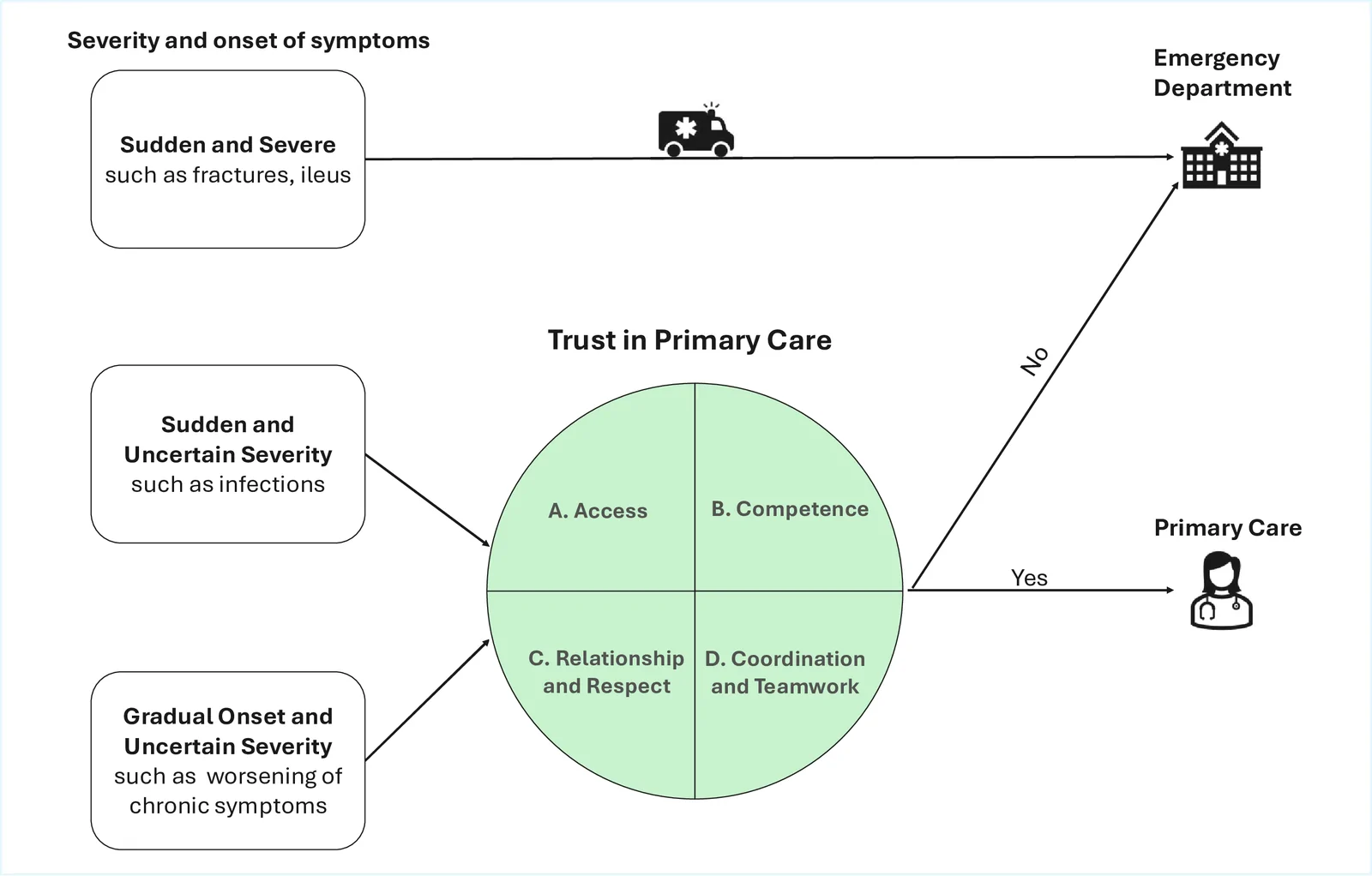
An illustration of the main theme and subthemes, and how we interpret them to influence care-seeking at the ED or HBPC. Main theme: Trust as a prerequisite for timely emergency assessments in home-based primary care. Subthemes: A: Accessible HBPC offering alternative care pathways to ED visits. B: Medical competence as a foundation for trust in HBPC. C: The importance of continuity and respect in patient-provider relationships. D: HBPC’s responsibility coordinate care through teamwork and follow-up. ED, emergency department; HBPC, home-based primary care.

Informants described several components of HBPC delivery that may help reduce ED visits. They were grouped into four subthemes–access, competence, respect and relationship and coordination and teamwork. When HBPC is accessible, provided by competent staff, characterised by respectful treatment and grounded in an established therapeutic relationship–within a coordinated, team-based model–older adults are more likely to develop trust in HBPC and to seek support from HBPC rather than the ED. Accordingly, the overarching theme can be conceptualised as *trust as a prerequisite for timely emergency assessments in HBPC* (figure 1).

### Overarching theme: Trust as a prerequisite for timely emergency assessments in HBPC

The informants’ reasons for ED visits were structured into three general groups: severe and sudden symptoms where ED care was the appropriate option, acute symptoms of uncertain urgency and gradually worsening symptoms over weeks or months (figure 1). Notably, informants across all three patterns described their ED visit as necessary and unavoidable. Only two informants receiving HBPC considered their ED visits avoidable: one suspected that a preceding medical error had caused the ED visit; the other had felt reluctant to contact HBPC. Some informants sought ED care for long-term symptoms or symptoms of uncertain severity without first contacting HBPC, while others described HBPC as their first line of contact. Still, most informants wished to avoid the ED, citing prolonged waiting times and insufficient assistance with basic needs during the wait.

Although informants had regular contact with HBPC staff, some felt that they did not receive adequate support from HBPC and expressed distrust in their general practitioner (GP). One family caregiver described this:

Family caregiver: And then you’re supposed to decide for yourself whether the morphine patch is a good idea or not, because that doctor - the secret doctor at the PC practice - you don’t know who it is, what kind of education they have, or whether they know anything about this. You really have no idea.

(Interview 13, female)

Not surprisingly, informants who distrusted their GP also expressed greater trust in the ED than in HBPC. In contrast, informants who expressed trust in HBPC preferred assessment by HBPC staff, even in emergency situations. The informants’ trust in HBPC appeared to influence their care-seeking behaviour, particularly when they experienced symptoms of uncertain urgency or gradual onset.

The following four subthemes describe the components of HBPC delivery interpreted as influencing trust in HBPC and potentially whether HBPC was used as an alternative to ED visits.

### Subtheme A: Accessible HBPC offering alternative care pathways to ED visits

If HBPC cannot be reached when symptoms arise, patients cannot trust it as an alternative to the ED. Informants described varying experiences of whether HBPC was accessible. Many emphasised that reaching HBPC and the PC practice should be simple and that rapid responses are crucial–for example, by allowing direct phone access that bypasses the regular patient queue. Informants also highlighted the importance of rapid assessments at home in emergency situations.

Patient: Then someone from HBPC came, she came in, just looked at me and said:” But dear, you can’t lie here like this. I see how bad it is.” Home care staff had called HBPC, and since then I’ve had HBPC. They arranged everything, and the next day I was picked up by medical transport and admitted to the geriatrics ward.

(Interview 5,patient: female)

One informant receiving HBPC, who had a history of heart disease and recurring chest pain, described with his daughter that they perceived GPs as unavailable for contact or emergency assessment. As a result, they considered calling an ambulance the only option in case of worsening symptoms, despite multiple prior ED visits that had not led to any medical intervention.

Patient: You can’t use the phone and call for a doctor; it’s only the ambulance. When the ambulance comes, we go to the hospital, you have to wait 10 hours before seeing a doctor. Is that good? You’re not well, and I don’t want to go to the hospital. Never. If you go to the PC practice and book an appointment, it’s not immediate - you have to wait a month to get one.

Family caregiver: He doesn’t want to go to the ED because he has to spend many hours there, several hours alone, and he’s already in pain when he arrives. Then he has to sit there uncomfortably - on hard beds and chairs - for several more hours. On top of that, he’s alone there and becomes even more anxious.

(Interview 6, patient: male, family caregiver: child of patient, female)

Outside regular office hours, HBPC was inaccessible, which caused frustration among informants. They expressed a wish to reach someone who is familiar with their medical history outside of office hours, rather than relying on ambulance services or the national healthcare advice line. Informants also highlighted the value of alternative care pathways to ED visits, such as direct admission to geriatric wards, same-day treatment at specialised heart failure centres or intermittent treatment by advanced home healthcare (Box 1) when intravenous drugs were needed.

### Subtheme B: Medical competence as a foundation for trust in HBPC

Informants emphasised that trust in HBPC depended not only on access to care but also on confidence in the medical competence of healthcare professionals. Some informants questioned the competence of GPs in assessing emergencies and doubted whether HBPC could provide appropriate care in urgent situations. In contrast, the ED was perceived as being staffed with competent professionals who could provide the necessary help, making it a potential shortcut to competent care. Consequently, some informants preferred the ED over HBPC and chose not to contact HBPC at all.

Patient: I wanted to take a shortcut to avoid going through the PC practice. That’s why I went to the ED. I thought that would be more efficient – that they’d send a direct referral.

Family caregiver: And it wasn’t as if the pain was constant, but at the moment the decision was made [to go to ED], it hurt more than usual. You felt like you had to do something to get answers. Otherwise, you wouldn’t have gone to the ED at all. You think: I can’t stand this, and who is going to do anything at the PC practice? They don’t know anything about this.

(Interview 13, patient: male, family caregiver: spouse of patient, female)

Relational continuity was valued only when the GP was perceived as medically competent. One informant receiving HBPC described how a pharmacist had identified a potentially serious drug-drug interaction and informed the GP, yet no change in treatment was made. The informant subsequently suffered a serious complication, which was attributed to the drug-drug interaction. Consequently, the informant considered the ED visit to be a result of medical misjudgement, which severely undermined trust in both this GP and HBPC in general.

Patient: I would have expected him [the GP] to prescribe a completely different dosage, and then what happened afterwards probably wouldn’t have occurred. So, the first visit to the hospital would have been unnecessary. And later, when I fell, I had to go back to the hospital again. So really, three hospital visits were unnecessary.

(Interview 2, patient: male)

### Subtheme C: The importance of continuity and respect in patient-provider relationships

Beyond medical competence, informants highlighted the importance of relational continuity, respect and being treated as a whole person. Informants highlighted that established relationships with healthcare professionals fostered feelings of safety and trust. Interprofessional teams, including physicians, registered nurses and nurse assistants, offered continuity of care and cultivated relationships in which informants felt genuinely respected and valued.

Family caregiver: Yes, they’re always there for you – they’re truly involved. They don’t just come and change the bandage and leave. They’re present. They are like family – that’s honestly how we feel. I’m so impressed, and happy.

(Interview 8, family caregiver: spouse of patient, female)

However, as their need for help with daily activities increased, some informants receiving HBPC described feeling infantilised and being assigned support they neither needed nor wanted. Instead, they wished to be treated as autonomous adults capable of making their own decisions and deserving dignity. Informants also noted that healthcare providers commonly focused on one problem or diagnosis at the time, while they wished to be considered whole persons.

Patient: A holistic view, yes. I think X, who was a bit like the GP I used to have, had a completely different approach. She had reviewed my medical records and seen what I had been through. She looks at the whole picture and tries to address any issues.

(Interview 2, patient: male)

Characteristics of a successful patient-GP relationship described by informants included having an assigned GP familiar with their medical history and feeling listened to during consultations. While some described a good relationship with their GP, others described a lack of relational continuity, with the GP perceived as a distant figure or even ‘a secret doctor’ as expressed by one informant, showing little interest in their patients. One patient described an anonymous encounter:

Patient: I think I have a female doctor, but I’m not sure.

Interviewer: Do you know when you last saw the doctor?

Patient: She came in, had a quick look at me and said:” No, that’s not necessary.”, and then she left.

Interviewer: Was this here, at home?

Patient: No, it was at the PC practice.

(Interview 14, patient: male)

Some informants with an assigned GP described that, in practice, their contact was limited to an annual evaluation, which they experienced as insufficient to develop a sense of continuity with their GP. They expressed a wish for more regular contact to ensure that their health concerns were addressed over time. This was interpreted as affecting perceived continuity and trust in the patient-GP relationship.

Patient: I said many times, I would like to see the doctor, and I want to… I want them to examine my heart, because I have heart failure. And I want them to check my blood pressure. [ ] It wouldn’t hurt (if the doctor would see me), and I think it’s been a year since last time.

(Interview 5, patient: female)

Informants who described high turnover of GPs at their PC practice particularly expressed low trust in HBPC and, in some cases, chose not to contact HBPC at all. For the individuals with negative experiences of the patient–GP relationship, the ED was often seen as a more reliable alternative to obtain care.

### Subtheme D: HBPC’s responsibility to coordinate care through teamwork and follow-up

In contrast to the trust or distrust in individual healthcare professionals described in Subtheme C, informants also described coordination, teamwork and follow-up by the HBPC organisation as central to trusting the organisation of HBPC. Consequently, this influenced whether they viewed HBPC as a reliable alternative to the ED.

Informants desired coordinated, consistent follow-up from HBPC. While some described that they received the care they needed, others reported contrasting experiences. HBPC was perceived as placing too much responsibility on patients, requiring them to initiate contact themselves. One informant receiving wound care at home thought that once her wounds were healed, no one would follow-up, leaving her with the responsibility to seek further contact with PC. As an alternative, informants expressed a wish for GPs to check in regularly without them having to initiate contact.

Family caregiver: There’s no one today like the English GP who goes around checking up on people. ”Oh, so your foot hurts today, but yesterday it was the heart.” Instead, you come in, and you mention one thing - it’s this or that - and they just focus on that one thing. No one asks ”How are you doing because of this?” or ”Do you want help with anything else?”.

(Interview 9, family caregiver: spouse of patient, female)

One informant even suggested implementing standardised visits for older adults at the age of 80, similar to regular health check-ups offered for children.

Family caregiver: You leave all older patients who don’t have specific needs alone. I mean, nobody comes to check if they have rugs lying around? No, I’m thinking of something like the 2-year check-up at the child health centre.

(Interview 12, family caregiver: child of patient, female)

Navigating the healthcare system was described as challenging due to limited collaboration and coordination, both across healthcare providers and between healthcare and social care. This lack of coordination was illustrated by informants’ descriptions of clear division between services.

Family caregiver: They [Home care services and HBPC] have stone walls - the Berlin wall - between them.

P: I don’t get the sense at all that those who come here know anything about my conditions.

(Interview 2, family caregiver: spouse of patient, female, patient: male)

Informants further described healthcare as fragmented, with the involvement of multiple professionals and an absence of a single, clearly responsible care provider. This fragmentation was perceived to have a negative impact on the quality of care, as described by one informant.

Patient: My overall experience of healthcare is that too many people are involved [ ]. The communication between those providing care and treatment… They don’t always seem to have good contact with earlier healthcare providers.

(Interview 3, patient: male)

In some cases, this fragmentation led informants to contact the ED instead of HBPC. For example, care plans were not always clear to the informants or forwarded to relevant caregivers.

Some informants perceived that they were cared for by a well-functioning team, while others experienced a lack of cooperation between professionals. The HBPC nurse was viewed by some as a key figure in coordinating care and serving as a link to their PC practice and the GP. Others felt that HBPC and the PC practice operated separately, despite being a part of the same organisation. Family caregivers felt burdened with coordinating medical treatments and healthcare visits. One family caregiver, who considered herself frail, worried about managing her husband’s care without support. The couple described it as an unattainable dream that HBPC nurses would communicate with the GP, and this lack of coordination ultimately led them to contact the ED.

## Discussion

This study explored how older adults receiving HBPC and their family caregivers experienced healthcare prior to an ED visit and how such visits may be reduced. The results suggest that a lack of trust in HBPC–both in the HBPC in general and in individual professionals–may hinder patients from seeking timely assessments, thereby limiting HBPC’s opportunity to reduce avoidable ED visits. Extending our initial expectation that access to timely in-home assessment would be important, our findings suggest that such access alone may be insufficient and is instead embedded in broader relational and organisational dimensions of trust in HBPC. Trust is strengthened by accessibility, medical competence, relational continuity, respect, coordinated care teams and consistent follow-up. Conversely, one informant vividly illustrated a lack of trust by describing a ‘secret doctor’–an anonymous physician of uncertain competence who failed to provide guidance within the complex healthcare system.

The informants expressed expectations of HBPC that included access to a reliable phone line, medically competent staff, a designated GP familiar with their medical history and support in coordinating their care. When these elements are lacking, patients may choose to not contact HBPC with symptoms of uncertain severity or gradual onset and instead seek care directly at the ED, even when receiving regular HBPC.

Our results align with previous qualitative studies conducted with older adults, which suggest that low trust in the GP influences decisions to visit ED.^9–11 19^ Accessibility and perceived competence shape patients’ trust in HBPC, compared with their trust in the ED as an institution.^9–11 19^ The idea that trust influences care-seeking behaviour is supported by a British survey where patients who reported low trust in their GP were significantly less likely to assess their ED visits as avoidable.^20^ Patients may hold different levels of trust–in the individual, the organisation and the broader system–in this case the healthcare professional, PC practice organising HBPC and healthcare system. In our analysis, trust in HBPC was interpreted by researchers as being related to having a familiar GP perceived as medically competent and treating patients with respect. This is consistent with previous research suggesting that caring and respectful relationships and perceived competence shape trust in GPs and PC, and that continuity can enhance trust when the GP is perceived as acting in the patient’s best interests.^21 22^

To design effective interventions, it is important to assess whether distrust stems from misconceptions–misplaced doubt in competent HBPC staff–or from valid concerns about poor-quality care.^23^ If HBPC is trustworthy but distrusted due to misconceptions, interventions may focus on changing patients’ attitudes through improved communication or relationship-building. However, if HBPC delivers poor-quality care, distrust is warranted and greater organisational changes are required to build trust. Our study examined informants’ perceptions rather than the actual trustworthiness of HBPC. Although general trust in HBPC may be difficult to influence through isolated interventions, accessible care provided by familiar caregivers could strengthen it.^22 23^

HBPC can potentially reduce ED visits,^3 4^ though the concept of HBPC is not clearly defined.^4 24^ Interdisciplinary teamwork, care coordination and rapid at-home assessments are recognised as potentially effective components of HBPC in reducing ED visits.^3 5^ Relationship continuity may also play an important role, as it is associated with better health outcomes and reduced ED use among older adults.^25 26^ Although most research focuses on the patient-physician relationship, patients receiving HBPC often interact primarily with nurses. However, the impact of nurse-patient continuity on healthcare use, including ED visits, remains insufficiently studied. Although nurse-led care continuity interventions for people with long-term conditions are associated with reduced hospitalisations,^27^ further research is needed to understand the impact of nurse-patient continuity in HBPC settings. Patients receiving HBPC are among the most vulnerable in PC,^6^ and it remains unclear to which extent nurse visits could be an alternative to GP visits in this group. In the current study, informants emphasised the importance of regular contact with their GP, who remains responsible for key medical decisions, such as medication adjustments and alternative care pathways to ED visits.

While relational continuity often refers to ongoing contact with the same GP,^28^ collaboration between the nurse and the GP in HBPC may serve as an alternative. Evidence from interdisciplinary teamwork in HBPC, as well as other interventions that reduce hospital use, highlights the importance of this shared continuity model.^3 4^ In this study, informants received HBPC, where GPs and registered nurses worked at the same PC practice, which offers opportunities for effective team coordination. Yet, despite this organisational structure, some informants perceived the GP as operating independently rather than as an integrated member of the HBPC team.

This observation aligns with another Swedish study that evaluated the effects of increasing physician access in home healthcare through a mobile team.^29^ While the frequency of physician visits increased, no reduction in ED visits or hospital admissions was observed, suggesting that simply increasing GP access is not sufficient.^29^ In contrast, a Swedish study on comprehensive geriatric assessment in PC, where nurses and GPs collaborated, showed reductions in both hospitalisation and ED visits.^30^ A French study on complex geriatric assessment in PC, comparing nurse-led and GP-led models, reported reduced hospital admissions only in the GP-led arm.^31^ The authors attributed the limited effect in the nurse-arm to insufficient collaboration between nurses and GPs, highlighting the importance of teamwork.^31^

### Strengths and limitations

This study included adults aged 65 and older receiving HBPC. Informants receiving HBPC could invite a family member or other support person to the interview, and half of them chose to do so. Family caregivers were included to enrich the data, assist with recollection of events, and provide support for informants during interviews. A methodological consideration is that interviews were conducted jointly with patients and family caregivers when caregivers were present. The perspectives of patients and family caregivers were analysed together, as they reflected shared experiences within the same care context rather than independent accounts. Joint interviews may have influenced what informants chose to disclose and how experiences were expressed. However, the perspectives of family caregivers were considered important, as they were often actively involved in care-related decision-making and healthcare contacts and therefore provided valuable insights into experiences of HBPC.

We used purposeful sampling^32^ of PC practices and patients to obtain diverse perspectives. However, practices with greater organisational or staffing challenges may be underrepresented, as the same constraints affecting service delivery may also have limited their ability to participate in research, which may affect the transferability of the findings. Recruitment was challenging, as several potential informants were too affected by disease to participate. Only one informant was born outside Sweden. Recruiting foreign-born individuals proved difficult, possibly due to language barriers. This may limit the transferability of our findings to foreign-born populations.

Additionally, we did not have access to medical records, and therefore background information on informants was limited to self-reported data. Consequently, information on the duration of HBPC prior to the ED visit was not available. This may have influenced informants’ opportunities to develop relationships with HBPC staff and could affect the transferability of the findings. We also lacked information on the number of medications, diagnoses, and level of cognitive impairment. We excluded patients with advanced cognitive impairment because they lacked the capacity to provide informed consent and to communicate their experiences in an interview. This should be considered when interpreting the results, as adults with advanced cognitive impairment are likely to be at even higher risk of ED visits. However, as all patients were receiving HBPC due to functional limitations and a need for regular healthcare at home, the sample is likely to represent a vulnerable older population with substantial care needs.

We chose to include informants who sought ED care for any reason, rather than pre-selecting potentially avoidable visits. This meant some visits were clearly unavoidable, but the approach ensured that urgent ED visits that might still have been prevented through effective PC–such as decompensated heart failure–were captured.

## Conclusion

Experiences of care before an ED visit, as reported by older adults receiving HBPC and their family caregivers, highlighted the central role of trust in HBPC when choosing whether to consult PC or the ED. Consequently, interventions aiming to reduce ED visits could focus on trust-building within HBPC. Trust appears to be strengthened through perceived accessibility, continuity in relationships with both GPs and nurses, visible collaboration within the care team and confidence in clinical competence. In HBPC, the potential for effective care coordination and close collaboration between nurses and GPs should be fully used and communicated to the patient.

## Supplementary material

10.1136/bmjopen-2026-118687 online supplemental file 1

## Data Availability

Data are available upon reasonable request.

## References

[1] Gruneir A, Silver MJ, Rochon PA (2011). Emergency department use by older adults: a literature review on trends, appropriateness, and consequences of unmet health care needs. Med Care Res Rev.

[2] Latham LP, Ackroyd-Stolarz S (2017). Defining potentially preventable emergency department visits for older adults. *IJH*.

[3] Pritchard C, Ness A, Symonds N (2020). Effectiveness of hospital avoidance interventions among elderly patients: A systematic review. CJEM.

[4] Zimbroff RM, Ornstein KA, Sheehan OC (2021). Home-based primary care: A systematic review of the literature, 2010-2020. J Am Geriatr Soc.

[5] Totten AM, White-Chu EF, Wasson N (2016). Home-based primary care interventions.

[6] Sundberg L, Sonde L, Johansson L (2024). Äldre som har både hemtjänst och hemsjukvård i stockholm – en registerstudie. Stiftelsen stockholms läns äldrecentrum. Report. https://aldrecentrum.se/enskilt-dokument/aldre-som-har-bade-hemtjanst-och-hemsjukvard-i-stockholm-en-registerstudie/.

[7] Martin FC, O’Halloran AM (2020). Tools for Assessing Frailty in Older People: General Concepts. Adv Exp Med Biol.

[8] Skivington K, Matthews L, Simpson SA (2021). A new framework for developing and evaluating complex interventions: update of Medical Research Council guidance. BMJ.

[9] Kolk D, Kruiswijk AF, MacNeil-Vroomen JL (2021). Older patients’ perspectives on factors contributing to frequent visits to the emergency department: a qualitative interview study. BMC Public Health.

[10] Goodridge D, Stempien J (2019). Understanding why older adults choose to seek non-urgent care in the emergency department: the patient’s perspective. CJEM.

[11] Lowthian JA, Smith C, Stoelwinder JU (2013). Why older patients of lower clinical urgency choose to attend the emergency department. Intern Med J.

[12] Braun V, Clarke V (2006). Using thematic analysis in psychology. Qual Res Psychol.

[13] Braun V, Clarke V (2021). One size fits all? What counts as quality practice in (reflexive) thematic analysis?. Qual Res Psychol.

[14] Sundquist K, Malmström M, Johansson SE (2003). Care Need Index, a useful tool for the distribution of primary health care resources. J Epidemiol Community Health.

[15] World Medical Association (2013). World Medical Association Declaration of Helsinki: Ethical Principles for Medical Research Involving Human Subjects. JAMA.

[16] Malterud K, Siersma VD, Guassora AD (2016). Sample Size in Qualitative Interview Studies: Guided by Information Power. Qual Health Res.

[17] Braun V, Clarke V (2022). Thematic analysis a practical guide.

[18] Tong A, Sainsbury P, Craig J (2007). Consolidated criteria for reporting qualitative research (COREQ): a 32-item checklist for interviews and focus groups. Int J Qual Health Care.

[19] Hallgren J, Ernsth Bravell M, Dahl Aslan AK (2015). In Hospital We Trust: Experiences of older peoples’ decision to seek hospital care. *Geriatric Nursing*.

[20] Calastri C, Buckell J, Crastes Dit Sourd R (2025). Avoidable visits to UK emergency departments from the patient perspective: A recursive bivariate probit approach. Health Policy.

[21] Tarrant C, Dixon-Woods M, Colman AM (2010). Continuity and trust in primary care: a qualitative study informed by game theory. Ann Fam Med.

[22] Merenstein Z, Shuemaker JC, Phillips RL (2023). Measuring Trust in Primary Care. Milbank Q.

[23] Taylor LA, Solomon MZ, Kaebnick GE (2023). Trust in Health Care and Science: Toward Common Ground on Key Concepts. *Hastings Center Report*.

[24] Genet N, Boerma WG, Kringos DS (2011). Home care in Europe: a systematic literature review. BMC Health Serv Res.

[25] Ionescu-Ittu R, McCusker J, Ciampi A (2007). Continuity of primary care and emergency department utilization among elderly people. Can Med Assoc J.

[26] Dyer SM, Suen J, Williams H (2022). Impact of relational continuity of primary care in aged care: a systematic review. BMC Geriatr.

[27] Davis KM, Eckert MC, Hutchinson A (2021). Effectiveness of nurse-led services for people with chronic disease in achieving an outcome of continuity of care at the primary-secondary healthcare interface: A quantitative systematic review. Int J Nurs Stud.

[28] Bazemore A, Merenstein Z, Handler L (2023). The Impact of Interpersonal Continuity of Primary Care on Health Care Costs and Use: A Critical Review. Ann Fam Med.

[29] Erwander K, Ivarsson K, Agvall B (2025). Association between a mobile team intervention in Swedish municipal home care and the effect on emergency department visits and hospitalizations among older adults. BMC Health Serv Res.

[30] Nord M, Lyth J, Alwin J (2021). Costs and effects of comprehensive geriatric assessment in primary care for older adults with high risk for hospitalisation. BMC Geriatr.

[31] Orcel V, Banh L, Bastuji-Garin S (2024). Effectiveness of comprehensive geriatric assessment adapted to primary care when provided by a nurse or a general practitioner: the CEpiA cluster-randomised trial. BMC Med.

[32] Palinkas LA, Horwitz SM, Green CA (2015). Purposeful Sampling for Qualitative Data Collection and Analysis in Mixed Method Implementation Research. Adm Policy Ment Health.

[33] Ludvigsson JF, Bergman D, Lundgren CI (2025). The healthcare system in Sweden. Eur J Epidemiol.

