# Supplementary material for: Qualitative study to explore which components of home-based primary care may reduce emergency department visits among older adults
Source: BMJ Open. 2026 Sep 11;16(9):e118687. doi: 10.1136/bmjopen-2026-118687 (PMC13583700; doi:10.1136/bmjopen-2026-118687)
Supplement: Supplementary file 1 [file bmjopen-16-9-s001.pdf]

# <u>Additional file 1. Interview guide</u>

- **Main questions in bold.**
    - Follow up questions only asked if relevant in plain text.
    - *Instructions to interviewer in italics.*

## Warm up

- When did you go to the emergency department?
- Which emergency department did you visit?
- Who recommended you should go to the emergency department?

## Main interview

- **Describe your healthcare contacts and how you experience them?**
- **What happened the day you last visited the emergency department?**
    - *If the visit wasn't clearly caused by symptoms of high urgency, focus on what the symtoms were like before the visit, what help they recieved before the visit, and what kind of help they sought themselves.*
    - Did the visit feel urgent to you, or had you had symptoms for a longer period of time?
    - What kind of help did you get at the emergency department? Who helped you?
    - Did you have any healthcare contacts before the emergency department visit? Did you have contact with home care, or a family member?
    - Did you feel satisfied with what happened before the emergency department visit? What were you satisfied with? What were you not satisfied with? Could you give concrete examples?
    - *If the patient had contact with healthcare before the visit.* What worked well with the care before the emergency department visit? What did not work well?
    - What was positive and negative with the emergency department visit?
    - Did you get the care you needed?
- **Do you think your emergency department visit could have been avoided and if so, how?**
    - What support had you needed to avoid the emergency department visit?
- **What happened when you got home?**
    - What could have helped your recovery?

    - What could help prevent future emergency department visit?

## Concluding questions

- What do you wish healthcare would do for you now?
- What did you think about participating in this interview?
- Do you have anything to add?
