# Supplementary material for: Qualitative study to explore which components of home-based primary care may reduce emergency department visits among older adults
Source: BMJ Open. 2026 Sep 11;16(9):e118687. doi: 10.1136/bmjopen-2026-118687 (PMC13583700; doi:10.1136/bmjopen-2026-118687)
Supplement: Supplementary file 2 [file bmjopen-2026-118687.reviewer_comments.pdf]

PEER REVIEW HISTORY

BMJ Open publishes all reviews undertaken for accepted manuscripts. Reviewers are asked to complete a checklist review form and are provided with free text boxes to elaborate on their assessment. These free text comments are reproduced below.

ARTICLE DETAILS

Title (Provisional)

A qualitative study to explore which components of home-based primary care may reduce emergency department visits among older adults

Authors

Bring, Hanna; Bergqvist, Monica; Gustafsson, Lars L; Modig, Karin; Bastholm-Rahmner, Pia; Schmidt-Mende, Katharina

---

VERSION 1 - REVIEW

**Reviewer**	**1**
**Name**	**Syed, Muslim**
**Affiliation**	**Primary Health Care Corporation**
**Date**	**12-Apr-2026**
**COI**	**None**

This is a timely and valuable qualitative study addressing an important question on how home-based primary care (HBPC) may reduce emergency department (ED) use among frail older adults. The manuscript would benefit from clearer methodological reporting and tighter alignment between the results and conclusions.

Methodology

Further detail on the interview process is needed, including who conducted the interviews, their relationship to participants, and measures taken to reduce interviewer bias.

It is advised, that the analytic approach would benefit from clearer description (e.g., coding framework, number of coders, how consensus was reached, and whether reflexivity was considered).

Results

The results section would be strengthened by more explicit linkage between themes, subthemes, and illustrative quotations to enhance transparency.

Some themes appear overlapping; clearer distinction and tighter framing would improve coherence.

It would be helpful to indicate whose perspectives (older adults vs. caregivers) are reflected in each theme where relevant.

Consider summarizing the key mechanisms by which HBPC reduces ED visits in a short integrative paragraph or figure to improve clarity.

Overall, with clearer methodological transparency and more structured presentation of findings, the study's contributions will be more convincing and easier to interpret for readers and policymakers.

Reviewer **2**

Name **Bula, Christophe**

Affiliation **University of Lausanne Medical Center (CHUV), Service of Geriatric Medicine & Geriatric Rehabilitation**

Date **19-May-2026**

COI **None**

General comments:

This qualitative study investigated the retrospective perceptions of home-based primary care (HBPC) among a small convenience sample of adults aged 65 years or more (N=14) who had been visiting an emergency department (ED) within the previous 3-month period in years 2022 to 2023 in Stockholm, Sweden. An additional sample of informal caregivers (N=7, whose relationship to the patients is not specified but seem to be their caregivers, see comments below) also provided information.

Results identified "Trust" as a major factor underlying decisions to use HBPC for an emergency assessment instead of going to the ED, with four dimensions (care access; medical competence; respect and relationship; coordination and team work) related to trust building toward HBPC.

The manuscript is very well written, the objectives are clearly stated. The Background section is clear and concise, it could benefit from the addition of some information on the authors' initial hypotheses (see specific comment below). The qualitative methodology is well described and appears appropriate. A question related to the relationship between participating patients and caregivers needs clarification (see comments below in Abstract and Methods sections). The presentation of the results is clear, well structured, and remain concise with well-chosen citations of participants. The discussion is appropriate.

As frequently in qualitative study, a significant concern relates to the selection process of study's participants. The issue of caregivers' relationships is of concern because if (as apparent in Results) they are related to some participants this raises the issue whether it is

appropriate to analyze their experience and assessments as if independent of the patient's ones. This needs to be clarified and discussed more extensively in the Discussion section.

The study certainly addresses an important concern of most health care systems and results confirm the importance of several dimensions that need specific attention to enhance the quality of home-based primary care. While some of the participants' comments reflect fully legitimate concerns (e.g., P13, L14-16: "... a wish to reach someone who is familiar with their medical history outside of office hours…") they unfortunately amount more to a wish list that will be tested by reality than to a realistic agenda. Whether addressing current other shortcomings identified in this study would truly reduce ED use will also need additional studies.

The study's topic is certainly relevant, but results from the small, very selected population are not really original. Overall, the reader remains undecided about the robustness and significance of its real contribution to this field of research.

Specific comments (CAVE: Page numbers are from authors numeration):

Title

1, P1, L7-8: The title seems somewhat misleading as it could be interpreted as if the study will provide direct solutions instead of information on patients and caregivers feelings about HBPC and ED use. Consider revising to something like : " A qualitative study to investigate dimensions of home-based primary care associated with potential reduction in emergency department visits by older adults."

In addition, I am also concerned by the use of "frail" in the Title (see specific comment below in Methods section).

Abstract:

2 P3, L14: I am not completely sure whether "aspects" is the most appropriate terminology. Consider replacing by "dimensions" or "characteristics".

3 P3, L37-39: Consider specifying already here whether these caregivers had any relationship with the N=14 participants. The formulation in P3, L13 ("and their informal caregivers…") and L23-24 ("..with their informal caregivers…) suggest this is the case, but no information is provided in the Methods section of the main text (see comment about the Methods section below). Indeed, it is only in Results (e.g., P11, L44: "..with his daughter…") that this becomes evident (correct?). This needs to be better explained and clarified. As already mentioned in the general comments, the implications for the analyses and results also needs to be discussed.

Strengths and limitations of this study:

4. P5, L17-28: An important limitation that needs to be mentioned is the exclusion of some older adults with cognitive impairment judged by research staff as precluding the interview. This seems particularly relevant as cognitive impairment has been associated with increased

risk of ED use, including inappropriate ED use. The authors mention that some participants might suffer from some level of cognitive impairment, but in the absence of objective measure and criteria, this remains a limitation and precludes conclusions about this specific population.

Background:

5. P7, L19-28: Based on the existing literature, the authors appropriately hypothesized that some ED visits could be prevented if the quality of HBPC were improved. It would be helpful for the reader if the authors could also share here some of their a priori hypotheses regarding the aspects/dimensions of care that they believed could be improved. Gaps in current literature about home-based care shortcomings could also already be mentioned here (even though they appear later in the Discussion section).

Methods:

6. P7, L8-14: The population selection process is, of course, extremely important in this qualitative study. If I am correct, only 11 of the 171 (6.4%) practices were able to identify participants. Could the authors provide more information on how these practices compare to those who did not provide patients? Besides the N=20 patients cut-off, was there any other inclusion/exclusion criteria applied?

7. P7, L8-14: The duration of home-care received by participants before the ED visit is certainly crucial for participants to build a relationship with the home-care staff and assess accessibility, competence, and ability to teamwork. Could the authors provide this information? If not, this should also be acknowledged as a limitation of the study.

8. P7, L8-14: The authors refer several times (e.g., in the Title and in the Introduction section) to the patients as “frail” older adults. Most readers would expect the use of some specific measure of frailty. However, apart from their age there is no specific information provided to support the use of this adjective. Even though the authors mention that HBPC patients are among the frailest primary care patients, the criteria to qualify for home-based primary care provided in Box 1 do not permit either the reader to firmly conclude about the patients’ level of frailty. Could the authors comment and, ideally, provide more information to support their perspective.

9. P7, L9-11: The authors should also provide some more information on the selection of informal caregivers. How were they identified? Were they (all? some?) involved in the care of the 14 patients who participated? What were the inclusion/exclusion criteria (if any)?

10. P8, L58: It seems somewhat surprising that the authors did not have some underlying conceptual framework/theory about home-care assessment by older adults and its relationship to potential ED overuse (see also comment above (about the Introduction section). Could the authors comment on this?

Results:

No specific comment.

Discussion:

11. P16, L21: I am not sure to agree with this sentence. For instance, the wish (P13, L14-16) “... a wish to reach someone who is familiar with their medical history outside of office hours…”) unfortunately does not seem modest to me, and I am afraid that most health care systems in western countries have difficulties to offer this level of continuity of care. Please consider revising.

References

12. Please verify and revise the following references that appear strange, with duplicate journal names, or incomplete: #1,#4,#5, #16, #22, #23, #30.

Tables

13. Table 1: As mentioned in a previous comment, it would be very informative to have some information about the duration of home-care prior to the ED event. If available, this information could be nicely incorporated in this Table 1.

Additional material

No specific comment.

VERSION 1 - AUTHOR RESPONSE

Reviewer: 1
Dr. Muslim Syed, Primary Health Care Corporation
Comments to the Author:

1. This is a timely and valuable qualitative study addressing an important question on how home-based primary care (HBPC) may reduce emergency department (ED) use among frail older adults. The manuscript would benefit from clearer methodological reporting and tighter alignment between the results and conclusions.

Authors’ response 1: Thank you for these positive comments and constructive suggestions. We agree that clearer methodological reporting and stronger alignment between the results and conclusions could strengthen the manuscript, and we have revised the manuscript according to the comments below.

2. Methodology
   Further detail on the interview process is needed, including who conducted the interviews, their relationship to participants, and measures taken to reduce interviewer bias.

Authors' response 2: Thank you for this comment. We agree that this information is important, and we would like to clarify that the manuscript already included details on who conducted the interviews, their experience and training, and the fact that interviewers had no established relationship with participants. We have now further clarified and expanded the description of reflexivity, particularly regarding how the interviewers reflected on their professional backgrounds and potential preconceptions during coaching and preparation to strengthen transparency in the interview process. When conducting reflexive thematic analysis according to Braun and Clarke, the interviewer's perspectives are not conceptualised as bias, but as an something that needs to be consider during the analytic process and something that inevitably shapes interpretation. From a constructivist standpoint, this influence is unavoidable; therefore, we avoid the term "interviewer bias" (Braun and Clarke 2021a).

Change to manuscript 2a:

Materials and Methods

Page 9, line 4-14

Two pilot interviews were conducted by co-author PBR in early 2022 and were included in the analysis. Following the pilot interviews, the interview guide was revised to place greater emphasis on the informants' experience during the period before the ED visit. Subsequent interviews were conducted by HB and IL between November 2022 and September 2023. PBR is an experienced qualitative researcher. HB and IL have experience working with frail older adults and were coached in qualitative interview techniques by PBR and MB. During interviewer coaching and preparation, the interviewers reflected on their interviewing technique and how their previous clinical experience and professional background could influence interactions with informants. This reflexive process was used to increase awareness of potential preconceptions. Interviewers had no previously established relationship with informants.

3. It is advised, that the analytic approach would benefit from clearer description (e.g., coding framework, number of coders, how consensus was reached, and whether reflexivity was considered).

Authors' response 3: Thank you for this comment. As stated in the results, HB conducted all coding and PBR, MB and KSM were all involved in further analysis. The analysis was inductive, meaning no pre-existing coding framework was applied. Instead, codes were developed iteratively from the data during analysis. The structure of the coding process is illustrated through the coding example provided in Table 2.

In reflexive thematic analysis according to Braun and Clarke, analysis is not based on a structured codebook approach, and analytic consensus is not the primary aim. Instead, multiple researchers' perspectives can be used to inform and deepen interpretation of the data (Braun and Clarke, 2021). The final analysis represents an interpretive account, with HB taking primary responsibility for the analytic process and final interpretation.

We already briefly discussed reflexivity in the Trustworthiness section but have expanded this section in the results.

Change to manuscript 3a:

Data analysis

Page 9, line 21-33

Inductive thematic analysis was conducted, following the six-step methodology outlined by Braun and Clarke.11 HB, PBR, and MB read all transcripts to become familiar with the data, made notes on patterns and discussed their observations. HB conducted the coding in NVivo (NVivo qualitative data analysis software, QSR International Pty Ltd, version 14), identifying similarities in the data and patterns of meaning without applying pre-existing theories or coding framework. After initial coding, the codes were examined and grouped into provisional themes based on shared meaning. For an example of coding, see table 2. Finally, HB, PBR, MB and KSM discussed and refined the salient patterns to enhance the depth of the analysis. These discussions were intended to support reflexive engagement with different interpretations and strengthen the analytic richness. HB was the main responsible researcher for the analysis and held final responsibility for the interpretation of the data, including in cases of differing analytic perspectives. ~~Participants~~ Informants were not invited to provide feedback on the findings.

Change to manuscript 3b:

Trustworthiness

Page 11, line 2-6

Confirmability was strengthened through ongoing discussions on reflexivity during analysis, considering how disciplinary backgrounds and assumptions shaped interpretations. For example, HB reflected on how her experience as a GP working with older adults in HBPC could influence data interpretation, while discussions within the research team helped challenge assumptions and broaden perspectives.

4. Results
   The results section would be strengthened by more explicit linkage between themes, subthemes, and illustrative quotations to enhance transparency.

Authors' response 4: Thank you for this comment. We have revised the Results section to improve clarity regarding the relationship between themes, subthemes, and illustrative quotations.

We describe the linkage between themes and subthemes in the first paragraph of the results section, and we made no changes to this section. We have added linking sentences in the end of the main theme summary and at the start of each subtheme. We have also reviewed the quotes, and where in one case we deemed the quote was insufficiently linked to the subtheme we have revised the linking sentence.

Result section describing linkage between themes and subthemes, no major changes.

Results

Page 11, line 8-15

Informants described several ~~aspects~~ components of HBPC delivery that may help reduce ED visits. They were grouped into four subthemes - access, competence, respect and relationship, and coordination and teamwork. When ~~home-based~~ HBPC is accessible, provided by competent staff, characterised by respectful treatment, and grounded in an established therapeutic relationship - within a coordinated, team-based model - older adults ~~are~~ may be more likely to develop trust in HBPC and to seek support from HBPC rather than the ED. Accordingly, the overarching theme can be conceptualised as trust as a prerequisite for timely emergency assessments in HBPC (Figure 1).

Change to manuscript 4a

Main theme

Page 12, line 10-12

The following four subthemes describe the components of HBPC delivery interpreted as influencing trust in HBPC and potentially whether HBPC was used as an alternative to ED visits.

Change to manuscript 4b:

Subtheme A

Page 12, line 14-15

If HBPC cannot be reached when symptoms arise, patients cannot trust it as an alternative to the ED.

Change to manuscript 4c:

Subtheme B

Page 13, line 17-18

Informants emphasized that trust in HBPC depended not only on access to care but also on confidence in the medical competence of healthcare professionals.

Change to manuscript 4d:

Subtheme C

Page 14, line 13-14

Beyond medical competence, informants highlighted the importance of relational continuity, respect and being treated as a whole person.

Change to manuscript 4e:

Subtheme D

Page 15, line 30-33

In contrast to the trust or distrust in individual healthcare professionals described in Subtheme C, informants also described coordination, teamwork and follow-up by the HBPC organisation as central to trusting the organisation of HBPC. Consequently, this influenced whether they viewed HBPC as a reliable alternative to the ED.

Change to manuscript 4f:

Subtheme C

Page 15, line 15-20

Some informants with an assigned GP described that, in practice, their contact was limited to an annual evaluation, ~~whereas they would have preferred more frequent contact to ensure their medical needs were monitored and addressed.~~ which they experienced as insufficient to develop a sense of continuity with their GP. They expressed a wish for more regular contact to ensure that their health concerns were addressed over time. This was interpreted as affecting perceived continuity and trust in the patient–GP relationship.

> *Patient: I said many times, I would like to see the doctor, and I want to… I want them to examine my heart, because I have heart failure. And I want them to check my blood pressure. [ ] It wouldn't hurt (if the doctor would see me), and I think it's been a year since last time. (Interview 5, patient: female)*

Results:

5. Some themes appear overlapping; clearer distinction and tighter framing would improve coherence.

Authors' response 5: Firstly, see comment 4, we have tightened the framing by adding framing sentences at the end of the main theme and start of each subtheme.

Secondly, we have carefully reviewed the results and found some overlap in the quotes and text of subtheme C. Of course, since all these concepts are closely related, there is some overlap in interpretation of quotes, and some quotes can contain more than one aspect. We have revised part subtheme C to more clearly

Change to manuscript 5a:

Subtheme C

Page 15, line 1-4

~~Informants identified several c~~Characteristics of a successful patient-GP relationship described by informants included~~:~~ having an assigned GP~~, the GP being available for short notice appointments, being~~ familiar with their medical history~~,~~ and feeling listened~~ing~~ to during consultations~~them~~.

Change to manuscript 5b:

See change to manuscript 4g

6. It would be helpful to indicate whose perspectives (older adults vs. caregivers) are reflected in each theme where relevant.

Authors' response 6: We thank the reviewer for the opportunity to clarify this. In our analysis, we did not systematically separate older adults and caregivers into distinct themes, but instead analysed the material as an integrated dataset, since many accounts were closely linked within interviews. However, we did distinguish perspectives where analytically relevant, particularly in relation to illustrative quotations and context-specific experiences (e.g. caregivers' experiences of burden in coordinating care). We have now clarified this approach in additional places in the manuscript to improve transparency.

Example of distinction in the original manuscript:

Results

Page 17, line 12-14

~~Informal~~ Family caregivers felt burdened with coordinating medical treatments and healthcare visits. One ~~informal~~ family caregiver, who considered herself frail, worried about managing her husband's care without support.

Change to manuscript 6a:

Results

Page 11, line 21

Only two informants receiving HBPC considered their ED visits avoidable

Change to manuscript 6b:

Page 13, line 33

Subtheme B:

One informant receiving HBPC described how a pharmacist had identified a potentially serious drug-drug interaction and informed the GP

7. Consider summarizing the key mechanisms by which HBPC reduces ED visits in a short integrative paragraph or figure to improve clarity.

Authors' response 7: We thank the reviewer for this suggestion. We agree that an integrative presentation of the mechanisms is important for clarity. We have already included such a synthesis in the opening section of the Results, where we summarise the four subthemes and explicitly describe how these relate to trust in HBPC and care-seeking between HBPC and the emergency department. This is further illustrated in Figure 1, which visualises the relationship between HBPC components, trust, and care-seeking pathways.

8. Overall, with clearer methodological transparency and more structured presentation of findings, the study's contributions will be more convincing and easier to interpret for readers and policymakers.

Authors' response 8: We have revised the manuscript to improve methodological transparency and to strengthen the structure and clarity of the findings.

Specifically, we have:

Added clearer framing sentences linking the subthemes to the overarching theme of trust in HBPC, see response comment 4.

Revised and refined the subthemes to better distinguish conceptual overlap, particularly between relational continuity (Subtheme C) and coordination of care (Subtheme D), response comment 5.

Clarified presentation of patient and caregiver perspectives throughout the Results section, response comment 6.

We believe these revisions improve the coherence of the analytic structure and make the interpretation of findings clearer for readers.

Reviewer: 2
Prof. Christophe Bula, University of Lausanne Medical Center (CHUV)
Comments to the Author:
General comments:
This qualitative study investigated the retrospective perceptions of home-based primary care (HBPC) among a small convenience sample of adults aged 65 years or more (N=14) who had been visiting an emergency department (ED) within the previous 3-month period in years 2022 to 2023 in Stockholm, Sweden. An additional sample of informal caregivers (N=7, whose relationship to the patients is not specified but seem to be their caregivers, see comments below) also provided information. Results identified "Trust" as a major factor underlying decisions to use HBPC for an emergency assessment instead of going to the ED, with four dimensions (care access; medical competence; respect and relationship; coordination and team work) related to trust building toward HBPC.

The manuscript is very well written, the objectives are clearly stated.

Authors' response 1: Thank you for this positive comment.

The Background section is clear and concise, it could benefit from the addition of some information on the authors' initial hypotheses (see specific comment below).

Authors' response 2: Thank you, we will respond to this under your comment number 5.

The qualitative methodology is well described and appears appropriate. A question related to the relationship between participating patients and caregivers needs clarification (see comments below in Abstract and Methods sections). The presentation of the results is clear, well structured, and remain concise with well-chosen citations of participants. The discussion is appropriate. As frequently in qualitative study, a significant concern relates to the selection process of study's participants. The issue of caregivers' relationships is of concern because if (as apparent in Results) they are related to some participants this raises the issue whether it is appropriate to analyze their experience and assessments as if independent of the patient's ones. This needs to be clarified and discussed more extensively in the Discussion section.

Authors' response 3: Thank you for this important reflection, we agree that it is important to clarify the relationship between informants and our analytic choices. Although informants were allowed to invite any family member, friend, or support person to the interview, all accompanying informants were in practice spouses or adult children who were involved in the patient's care to varying degrees. We have revised the manuscript to clarify this. We have changed the term from informal caregivers to family caregivers to clarify the relationship. Additionally, we have also clarified the relationship between the patient and family caregiver throughout the Results section and in the presentation of quotations.

Interviews were conducted with patients and, when present, family caregivers together in the same interview setting. Family caregivers were included as informants in their own right, and their perspectives were analysed alongside those of patients. We did not treat caregiver accounts as independent cases separate from the patient, but as co-constructed perspectives within the same care context. Both perspectives were therefore considered analytically valuable in understanding experiences of home-based primary care.

We acknowledge that joint interviews may have influenced how perspectives were expressed and what was shared. At the same time, including caregivers was an intentional design choice to capture experiences of care in a context where family caregivers are often actively involved in decision-making and care navigation for frail older adults. This was briefly discussed as a limitation, and we have now expanded that discussion.

Change to manuscript 3a:

Materials and Methods

Page 7, line 19-26

Fourteen patients registered with 11 PC practices were interviewed in their homes. Patients could invite a family member or other support person to participate in the interview, and; seven ~~interviews~~ chose to do so ~~also included an informal caregiver~~, resulting in a total of 21 informants. Although inclusion was not restricted to individuals with caregiving roles, all accompanying persons were spouses or adult children and were involved in the patient's care to varying degrees. For clarity, these informants are referred to as family caregivers in the results and discussion.

Change to manuscript 3b

Discussion:

Page 19, line 27 - page 20, line 9

This study included ~~frail~~ adults aged 65 and older receiving HBPC. Informants receiving HBPC could invite a family member or other support person to the interview, and half of them chose to do so. ~~Informal~~ Family caregivers were included to enrich the data, assist with recollection of events, and ~~help~~ provide support for informants ~~receiving HBPC feel safe~~ during interviews. ~~A possible concern when including informal caregivers is that they might dominate the conversation or prioritize their own perspective. However, this was not observed during our interviews. Overall, the benefits of including informal caregivers were judged to outweigh the potential disadvantages.~~ A methodological consideration is that interviews were conducted jointly with patients and family caregivers when caregivers were present. The perspectives of patients and family caregivers were analysed together, as they reflected shared experiences within the same care context rather than independent accounts. Joint interviews may have influenced what informants chose to disclose and how experiences were expressed. However, the perspectives of family caregivers were considered important, as they were often actively involved in care-related decision-making and healthcare contacts, and therefore provided valuable insights into experiences of HBPC.

The study certainly addresses an important concern of most health care systems and results confirm the importance of several dimensions that need specific attention to enhance the quality of home-based primary care. While some of the participants' comments reflect fully legitimate concerns (e.g., P13, L14-16: "... a wish to reach someone who is familiar with their medical history outside of office hours…") they unfortunately amount more to a wish list that will be tested by reality than to a realistic agenda. Whether addressing current other shortcomings identified in this study would truly reduce ED use will also need additional studies.

Authors' response 4: We agree that this study cannot determine whether addressing the identified shortcomings would lead to a reduction in emergency department visits. Rather, the aim was to explore how older adults and their family members perceived home-based

primary care and which aspects of care they considered important when deciding whether to seek support from home-based primary care or the emergency department.

We also agree that the effectiveness of interventions targeting these issues must be evaluated empirically. This study forms part of a larger research project on reducing emergency department visits among older adults. Together with findings from our studies of healthcare and social care staff, the present findings have informed an ongoing co-design process involving patients and healthcare professionals. This work focuses on developing and testing interventions to improve integration between health and social care and access to timely home-based assessments for frail older adults.

The study's topic is certainly relevant, but results from the small, very selected population are not really original. Overall, the reader remains undecided about the robustness and significance of its real contribution to this field of research.

Authors' response 5: We appreciate the reviewer's thoughtful comment regarding originality, sample size, and contribution. Our study was not intended to produce statistically generalizable findings, but to provide an in-depth qualitative understanding of how older patients experience home-based primary care. In qualitative research, the value of a study lies in the richness of the data, the transparency of the analytic process, and the relevance of the insights for theory and practice rather than in sample size alone.

We address the points in turn.

Sample size and study population
We included 21 informants (14 patient cases), which is within the range commonly considered appropriate for qualitative interview studies. In line with principles of information power, data collection was guided by the richness and relevance of the material rather than sample size alone and was concluded when sufficient depth and variation had been achieved to address the study aim (Braun and Clarke 2021b; Malterud et al. 2016). While the number of cases may be considered modest, this is consistent with qualitative methodology, where the aim is not statistical generalizability but in-depth understanding of experiences and perspectives.

Selection of informants
The study used purposive sampling to recruit informants receiving HBPC, in line with the study aim. Efforts were made to ensure variation in geographical and socio-economic context. As noted in the manuscript, we experienced challenges recruiting informants born outside Sweden, which is acknowledged as a limitation. We believe the sample nevertheless provides relevant insight into experiences of home-based primary care in the Swedish context.

Originality and contribution
We acknowledge that several of the identified issues have been described in previous research across different healthcare settings. However, our study contributes by exploring these

experiences specifically within the context of home-based primary care for older adults in a Scandinavian setting. In Sweden, primary care and home healthcare are jointly organized, which is not commonly the case. Our findings highlight that integrating home healthcare into primary care alone may not be sufficient to ensure coordinated and accessible care.

Specific comments (CAVE: Page numbers are from authors numeration):

Title

1, P1, L7-8: The title seems somewhat misleading as it could be interpreted as if the study will provide direct solutions instead of information on patients and caregivers feelings about HBPC and ED use. Consider revising to something like : " A qualitative study to investigate dimensions of home-based primary care associated with potential reduction in emergency department visits by older adults."

Authors' response 6: you for this insightful suggestion, we have changed the title of our manuscript, inspired by your suggestion.

Change to title:

A qualitative study to explore which components of home-based primary care may reduce emergency department visits among older adults

In addition, I am also concerned by the use of "frail" in the Title (see specific comment below in Methods section).

Authors' response 7: Thank you, for further detail see our response to your comment number 8, our response number 14, in the methods section. We have changed the title, see comment above.

2 P3, L14: I am not completely sure whether "aspects" is the most appropriate terminology. Consider replacing by "dimensions" or "characteristics".

Authors' response 8: Thank you for this comment. In the research group there was extensive discussion on which term would be most appropriate. We appreciate your suggestions and have chosen to change aspects to components.

Change to manuscript 8a:

Abstract

Page 3, line 7-8

to identify ~~aspects~~ components of home-based primary care delivery that could help reduce such visits.

Change to manuscript 8b:

Background

Page 7, line 2-3

to identify ~~aspects~~ components of HBPC delivery

Change to manuscript 8c:

Results

Page 11, line 8-9

Informants described several ~~aspects~~ components of HBPC delivery that may help reduce ED visits.

3 P3, L37-39: Consider specifying already here whether these caregivers had any relationship with the N=14 participants. The formulation in P3, L13 ("and their informal caregivers…") and L23-24 ("..with their informal caregivers…) suggest this is the case, but no information is provided in the Methods section of the main text (see comment about the Methods section below). Indeed, it is only in Results (e.g., P11, L44: "..with his daughter…") that this becomes evident (correct?). This needs to be better explained and clarified. As already mentioned in the general comments, the implications for the analyses and results also needs to be discussed.

Authors' response 9: Thank you for this comment, this is an important clarification to make. We have elaborated on this in the methods section and strengths and limitations section. See **Authors' response 3**.

4. P5, L17-28: An important limitation that needs to be mentioned is the exclusion of some older adults with cognitive impairment judged by research staff as precluding the interview. This seems particularly relevant as cognitive impairment has been associated with increased risk of ED use, including inappropriate ED use. The authors mention that some participants might suffer from some level of cognitive impairment, but in the absence of objective measure and criteria, this remains a limitation and precludes conclusions about this specific population.

Authors' response 10: We thank the reviewer for this important comment. We agree that cognitive impairment is highly relevant in this population.

We first respond to the comment about objective measure and criteria. Patients receiving home-based primary care often have varying degrees of cognitive and physical impairment. As we did not have access to medical records and cognitive impairment is frequently underdiagnosed in routine care, we had no objective measure of cognitive status. We did not consider perform cognitive testing on patients, as this would not be ethically appropriate or necessary for the aims of the study.

Secondly, we respond to the exclusion of some informants with advanced cognitive impairment. We did not use cognitive impairment as an exclusion criterion. However, we did consider cognitive status carefully during recruitment and data collection. Since we had no objective measures, recruitment was supported by clinicians working closely with the patients, who helped assess whether individuals were able to provide informed consent and participate in an interview. We also allowed informants to include a family member or support person in the interview to facilitate participation and support recall of events. We made this methodological choice partly because participation required the capacity to provide informed consent and partly because individuals with advanced cognitive impairment may have difficulty reflecting on and elaborating their experiences in an interview setting.

We acknowledge this as a limitation, and we have added clarification in the Discussion that the absence of objective cognitive assessment and medical record data may affect transferability and limits conclusions regarding patients with more severe cognitive impairment who were not included. We have also added a sentence and reference about the general level of cognitive impairment in this population in the background.

Change to manuscript 10a:
Background

Page 6, line 8-9

More than half of patients receiving both home care and HBPC have cognitive impairment,

Change to manuscript 10b:

Discussion:

Page 20, line 18-19

Additionally, we did not have access to medical records, and therefore background information on informants was limited to self-reported data.

Change to manuscript 10c:

Discussion:

Page 20, line 22-29

We also lacked information on number of medications, diagnoses, and level of cognitive impairment. However, as all patients were receiving HBPC due to functional limitations and a need for regular healthcare at home, the sample is likely to represent a frail older population with substantial care needs. We excluded patients with advanced cognitive impairment because they lacked the capacity to provide informed consent and to communicate their experiences in an interview. This should be considered when interpreting the results, as adults with advanced cognitive impairment is likely to be at even higher risk of ED visits.

5. P7, L19-28: Based on the existing literature, the authors appropriately hypothesized that some ED visits could be prevented if the quality of HBPC were improved. It would be

helpful for the reader if the authors could also share here some of their a priori hypotheses regarding the aspects/dimensions of care that they believed could be improved. Gaps in current literature about home-based care shortcomings could also already be mentioned here (even though they appear later in the Discussion section).

Authors' response 11: We thank the reviewer for this helpful suggestion. We have now clarified our a priori expectations and relevant literature in the Background section and added a linking interpretation in the Discussion.

Change to manuscript 11a:

Background

Page 6, line 18-23

Based on existing literature on HBPC, interdisciplinary teamwork, care coordination, and rapid at-home assessment have been suggested as potentially important components in reducing ED visits.[3,5] Drawing on this knowledge and clinical experience of HBPC in Stockholm, we expected that in particular limited access to timely in-home assessment, might be an important factor influencing care-seeking prior to ED visits.

Change to manuscript 11b:

Discussion

Page 17, line 22-25

Extending our initial expectation access to timely in-home assessment would be important, our findings suggest that such access alone may be insufficient and is instead embedded in broader relational and organisational dimensions of trust in HBPC.

Methods:

6. P7, L8-14: The population selection process is, of course, extremely important in this qualitative study. If I am correct, only 11 of the 171 (6.4%) practices were able to identify participants. Could the authors provide more information on how these practices compare to those who did not provide patients? Besides the N=20 patients cut-off, was there any other inclusion/exclusion criteria applied?

Authors' response 12: Regarding the participating primary care practices, as this is a qualitative study, our aim was not to achieve statistical representativeness of all 171 practices, but to obtain a purposive sample with variation in relevant contextual factors. Recruitment was therefore guided by geographical distribution and the Care Need Index (CNI), a deprivation index, in order to capture variation in socioeconomic conditions across practices.

All eligible practices were contacted via email or telephone, and the only inclusion criterion was the availability of at least 20 eligible HBPC patients, as smaller practices were considered unlikely to identify cases. No additional criteria were applied.

We acknowledge that recruitment may have influenced the transferability of findings, as practices with the greatest staffing and organisational challenges may have been less able to participate due to the same constraints affecting both service delivery and capacity to engage in research. We have added a sentence in the Discussion to clarify this and to contextualise the study sample

Change to manuscript 12:

Discussion,

Page 20, line 10-13

We used purposeful sampling[33] of PC practices and patients to obtain diverse perspectives. However, practices with greater organisational or staffing challenges may be underrepresented, as the same constraints affecting service delivery may also have limited their ability to participate in research, which may affect the transferability of the findings.

7. P7, L8-14: The duration of home-care received by participants before the ED visit is certainly crucial for participants to build a relationship with the home-care staff and assess accessibility, competence, and ability to teamwork. Could the authors provide this information? If not, this should also be acknowledged as a limitation of the study.

Authors' response 13: Thank you for this comment. We agree that the duration of HBPC prior to the emergency department visit is an important contextual factor. Unfortunately, this information was not collected in the study, and we did not have access to informants' medical records due to ethical approval limitations. We have therefore added this as a limitation in the Discussion section, noting that this may influence the transferability of the findings.

Change to manuscript 13:

Discussion

Page 20, line 18-22

Additionally, we did not have access to medical records, and therefore background information on informants was limited to self-reported data. Consequently, information on the duration of HBPC prior to the emergency department visit was not available. This may have influenced informants' opportunities to develop relationships with HBPC staff and could affect the transferability of the findings.

8. P7, L8-14: The authors refer several times (e.g., in the Title and in the Introduction section) to the patients as "frail" older adults. Most readers would expect the use of some specific measure of frailty. However, apart from their age there is no specific information provided to support the use of this adjective. Even though the authors mention that HBPC patients are among the frailest primary care patients, the criteria to qualify for home-based primary care provided in Box 1 do not permit either the reader to firmly conclude about the patients' level

of frailty. Could the authors comment and, ideally, provide more information to support their perspective.

Authors' response 14: Thank you for this important comment and for the opportunity to clarify our use of the term "frail".

We agree that we did not formally assess frailty using a validated frailty instrument in this study. The term "frail" was therefore not used as a measured variable, but as a descriptive characterization of the population based on the nature of who is eligible for HBPC and prior knowledge of this patient group.

HBPC in the Stockholm setting is provided to older adults with substantial health and functional limitations, typically including high levels of dependency in activities of daily living and a high prevalence of cognitive impairment. To support this contextual description, we have added a reference to regional data on HBPC and home care populations in Stockholm, which demonstrates high levels of functional dependency and cognitive impairment, although without a formal frailty index.

In response to the reviewer's comment, we have revised the manuscript to reduce reliance on the term "frail" where it could be interpreted as frail according to a formal frailty assessment and instead describe the population in terms of observed care needs, functional dependency, and cognitive impairment, added a reference, or in some cases removed the word altogether.

Change to manuscript 14a:

Abstract

Page 3, line 29 - page 4, line 1

Trust in both home-based primary care in general and in individual healthcare professionals is crucial in reducing emergency department visits among ~~frail~~ older adults receiving home-based primary care.

Change to manuscript 14b:

Background

Page 6, line 6-10

The population receiving HBPC in Stockholm consists of individuals with significant functional limitations and a need for support from healthcare services due to difficulties leaving their homes.[6] More than half of patients receiving both home care and HBPC have cognitive impairment, and approximately 85% require assistance with at least one activity of daily living, indicating a high level of frailty in this population.[6,7]

Change to manuscript 14c:

Background

Page 6, line 15-18

The experiences of older adults with a high degree of ~~frailty~~functional and cognitive impairment, such as those receiving HBPC[6], are less well understood, despite their greater care needs and frequent contact with healthcare providers.

Change to manuscript 14d:

Materials and Methods

Page 8, line 10-11

Older adults receiving HBPC ~~are a frail population and~~ often experience cognitive impairment.[6]

Change to manuscript 14e:

Discussion

Page 18, line 1-3

When these elements are lacking, ~~frail older adults~~ patients may choose to not contact HBPC with symptoms of uncertain severity or gradual onset

Change to manuscript 14f:

Discussion

Page 19, line 4

Patients receiving HBPC are among the frailest in PC[6]

Change to manuscript 14g:

Discussion

Page 19, line 27

This study included ~~frail~~ adults aged 65 and older receiving HBPC

9. P7, L9-11: The authors should also provide some more information on the selection of informal caregivers. How were they identified? Were they (all? some?) involved in the care of the 14 patients who participated? What were the inclusion/exclusion criteria (if any)?

Authors' response 15: Thank you for this comment. Patients decided whether they wished to include a family member or other person for support during the interview. There were no predefined inclusion or exclusion criteria for informal (family) caregivers. Caregiving was not an inclusion criterion. However, in practice, all accompanying persons were spouses or children, and they were involved in the care of the patient to varying degrees.

Family caregivers were included as informants when present, as they were often involved in care-related decision-making and contact with healthcare services and could provide complementary perspectives on the patients' care experiences. We have clarified this

procedure and revised the wording in the Methods section for consistency, see **Authors' response 3.**

10. P8, L58: It seems somewhat surprising that the authors did not have some underlying conceptual framework/theory about home-care assessment by older adults and its relationship to potential ED overuse (see also comment above (about the Introduction section). Could the authors comment on this?

Authors' response 16: Thank you for this thoughtful comment. We agree that conceptual frameworks can be valuable for understanding healthcare utilisation and emergency department use among older adults.

However, this study was designed as an exploratory qualitative study using an inductive approach. At the time of study design, there was limited knowledge regarding how older adults receiving home-based primary care and their family caregivers experience care in the period preceding an emergency department visit. We therefore chose not to apply a predefined conceptual framework, as our aim was to allow informants' experiences and perspectives to guide the analysis rather than interpret the data through an existing theoretical lens.

That said, our findings resonate with concepts described in previous literature on healthcare utilisation, including the importance of trust, accessibility, continuity, and care coordination in shaping care-seeking behaviour. We have clarified this rationale in the background section.

Change to manuscript 16:

Background

Page 6, line 24-25

Given the limited conceptual clarity in this specific context, the study adopted an exploratory qualitative design, allowing informants' experiences to guide the analysis.

Results:
No specific comment.

Discussion:
11. P16, L21: I am not sure to agree with this sentence. For instance, the wish (P13, L14-16) "... a wish to reach someone who is familiar with their medical history outside of office hours…") unfortunately does not seem modest to me, and I am afraid that most health care systems in western countries have difficulties to offer this level of continuity of care. Please consider revising.

Authors' response 17: Thank you for this comment. Our intention in this paragraph was to highlight the more basic or fundamental expectations of HBPC that informants described as important for receiving and engaging with care. However, we agree that the term "modest expectations" may be misleading, particularly given that some expectations (e.g. continuity

outside office hours) may be more demanding in practice. We have therefore removed this phrasing and revised the sentence to more neutrally describe the expectations expressed by informants.

Change to manuscript 17:

Discussion

Page 17, line 30 – page 18, line 1

~~The informants expressed what could be considered modest expectations of HBPC. They wanted~~ The informants expressed expectations of HBPC that included access to a reliable phone line, medically competent staff, a designated GP familiar with their medical history, and support in coordinating their care.

References

12. Please verify and revise the following references that appear strange, with duplicate journal names, or incomplete: #1,#4,#5, #16, #22, #23, #30.

Authors' response 18: Thank you for bringing this to our attention, we have revised the references.

Tables

13. Table 1: As mentioned in a previous comment, it would be very informative to have some information about the duration of home-care prior to the ED event. If available, this information could be nicely incorporated in this Table 1.

Authors' response 19: We agree that this information is important and would fit in Table 1, but unfortunately, we do not have access to that information, see comment 7, Authors' response 13.

References

Braun, Virginia, and Victoria Clarke. 2021a. "One Size Fits All? What Counts as Quality Practice in (Reflexive) Thematic Analysis?" *Qualitative Research in Psychology* 18 (3): 328–52. https://doi.org/10.1080/14780887.2020.1769238.

Braun, Virginia, and Victoria Clarke. 2021b. "To Saturate or Not to Saturate? Questioning Data Saturation as a Useful Concept for Thematic Analysis and Sample-Size Rationales." *Qualitative Research in Sport, Exercise and Health* 13 (2): 201–16. https://doi.org/10.1080/2159676X.2019.1704846.

Malterud, Kirsti, Volkert Dirk Siersma, and Ann Dorrit Guassora. 2016. "Sample Size in Qualitative Interview Studies: Guided by Information Power." *Qualitative Health Research* (Thousand Oaks) 26 (13): 1753–60. https://doi.org/10.1177/1049732315617444.

VERSION 2 - REVIEW

Reviewer 2

Name Bula, Christophe

Affiliation University of Lausanne Medical Center (CHUV), Service of Geriatric Medicine & Geriatric Rehabilitation

Date 28-Jun-2026

COI

Thank you for your detailed revision of the paper. I have only few additional comments.

Strengths and limitations

1, P38: I would strongly recommend to reintroduce the following bullet point as it acknowledges a clear and important limitation of the study. If necessary, the sentence can be shortened by deleting the part after "sampling".

"Informants were identified by home-based primary care staff, which may have affected sampling if staff favoured recruitment of individuals with certain characteristics or perspectives."

2. Consider also adding a bullet point about the exclusion of informants with advanced (moderate to severe?) cognitive impairment.

Background and Discussion:

2 P6, L10: Thank you for revising the use of the term "frail" and "frailty". Consider replacing "frail" and "frailty" by "vulnerable" and "vulnerability" throughout the manuscript to avoid any confusion with the frailty concept.

VERSION 2 - AUTHOR RESPONSE

Reviewer: 2
Prof. Christophe Bula, University of Lausanne Medical Center (CHUV)
Comments to the Author:
Thank you for your detailed revision of the paper. I have only few additional comments.

Authors' response: We thank the reviewer for taking the time to once more review our revised manuscript and for the additional helpful comments. We have carefully considered each of the comments and addressed them below. Please note that we refer to our own page and line numbering throughout the responses.

Strengths and limitations

1, P38: I would strongly recommend to reintroduce the following bullet point as it acknowledges a clear and important limitation of the study. If necessary, the sentence can be shortened by deleting the part after "sampling".
"Informants were identified by home-based primary care staff, which may have affected sampling if staff favoured recruitment of individuals with certain characteristics or perspectives."

Authors' response: Thank you for this suggestion, the bullet-point has been reintroduced according to your suggestion but slightly shortened.

page 5, line 12-13

- Informants were identified by home-based primary care staff, which may have influenced participant selection and the perspectives included.

2. Consider also adding a bullet point about the exclusion of informants with advanced (moderate to severe?) cognitive impairment.

Authors' response: Thank you. We have added a bullet point addressing the exclusion of informants unable to provide informed consent or participate in an interview.

page 5, line 15-17

- Informants unable to provide informed consent or participate in an interview due to cognitive impairment were excluded, which may limit the transferability.

Background and Discussion:
2 P6, L10: Thank you for revising the use of the term "frail" and "frailty". Consider replacing "frail" and "frailty" by "vulnerable" and "vulnerability" throughout the manuscript to avoid any confusion with the frailty concept.

Authors' response: Thank you for this thoughtful suggestion, we agree that vulnerability is a more suitable concept in our manuscript and have changed this throughout the manuscript.

Change 1:

page 3, line 3-4

Home-based primary care has been suggested to reduce emergency department visits among ~~frail~~vulnerable older adults

Change 2:

page 5, line 20-21

~~Frail~~ Vulnerable older adults are more likely to visit the emergency department (ED) than the general population

Change 3:

page 5, line 26-28

Home-based primary care (HBPC) - also referred to as home healthcare in Scandinavian countries (box 1) - has in the US shown potential to reduce ED visits among ~~frail~~vulnerable, older adults with difficulties leaving their home due to illness or functional limitations.[3,4]

Change 4:

page 5, line 37 - page 6, line 3

More than half of adults receiving both home care and HBPC have cognitive impairment, and approximately 85% require assistance with at least one activity of daily living, indicating a high level of ~~frailty~~ vulnerability in this population.[6,7]

Change 5:

page 9, line 1-2

HB and IL have experience working with ~~frail~~ vulnerable older adults and were coached in qualitative interview techniques by PBR and MB.

Change 6:

page 18, line 25-26

Patients receiving HBPC are among the ~~frailest~~ most vulnerable in PC[6]

Change 7:

page 20, line 17-19

However, as all patients were receiving HBPC due to functional limitations and a need for regular healthcare at home, the sample is likely to represent a ~~frail~~ vulnerable older population with substantial care needs.

Reviewer: 2

If you have selected 'Yes' above, please provide details of any competing interests.: NA
